# Asymptomatic Severe and Moderate Aortic Stenosis: Time for Appraisal of Treatment Indications

**DOI:** 10.1016/j.shj.2023.100201

**Published:** 2023-06-27

**Authors:** Marko Banovic, Bernard Iung, Wojtek Wojakowski, Nicholas Van Mieghem, Jozef Bartunek

**Affiliations:** a Belgrade Medical Faculty, University of Belgrade, Belgrade, Serbia; b Cardiology Department, University Clinical Center of Serbia, Belgrade, Serbia; c University of Paris, Paris, France; d Department of Cardiology, Assistance Publique-Hôpitaux de Paris (AP-HP), Bichat Hospital, Paris, France; e Department of Cardiology and Structural Heart Diseases, Medical University of Silesia, Katowice, Poland; f Thoraxcenter, Erasmus University Medical Center, Rotterdam, the Netherlands; g Cardiovascular Center Aalst, OLV Hospital, Aalst, Belgium

**Keywords:** Aortic stenosis, Asymptomatic, Moderate, Treatment

## Abstract

Over the last decades, we have witnessed considerable improvements in diagnostics and risk stratification of patients with significant aortic stenosis (AS), paralleled by advances in operative and anesthetic techniques. In addition, accumulating evidence points to the potential benefit of early valve replacement in such patients prior to the onset of symptoms. In parallel, interventional randomized trials have proven the benefit of transcatheter aortic valve replacement in comparison to a surgical approach to valve replacement over a broad risk spectrum in symptomatic patients with AS. This article reviews contemporary management approaches and scrutinizes open questions regarding timing and mode of intervention in asymptomatic patients with severe AS. We also discuss the challenges surrounding the management of symptomatic patients with moderate AS as well as emerging dilemmas related to the concept of a life-long treatment strategy for patients with AS.

Valvular intervention in patients with aortic stenosis (AS) is traditionally considered only if patients present with hemodynamically severe AS associated with clinical symptoms. The prognostic significance of asymptomatic significant AS or prognostic implication of earlier stages is less understood. Consequently, clinical practice and professional guidelines advise watchful waiting for such patients with regular follow-up.

Growing clinical experience and trial evidence are supporting the breakthrough of transcatheter aortic valve replacement (TAVR) as a viable treatment option as compared to surgical aortic valve replacement (SAVR) in symptomatic patients with significant AS. In addition, the notion of an adverse impact of severe AS on clinical outcome and cardiac structure, even in the absence of overt clinical symptoms, appears to be influencing clinical practice such that the timing of SAVR is becoming more liberal in a subset of patients with critical AS who until recently would not have been considered for intervention. This dynamic clinical landscape seems to be shifting the frontier of AS treatment towards earlier stages, as attested by changes in thresholds of AS severity and left ventricular ejection fraction (LVEF) over time in guidelines for intervention in asymptomatic AS. On the other hand, appropriate life-long AS treatment strategy is emerging as a dilemma, particularly in younger patients, posing a series of challenging decisions not only in the heart team's considerations but also in patient and family shared decision-making in devising the optimal, patient-tailored strategy.

In this review, we address contemporary management approaches and scrutinize open questions regarding timing and mode of intervention in asymptomatic patients with severe AS, including low-flow AS. We also address challenges surrounding the management of patients with moderate AS and open questions related to the concept of life-long strategy for AS treatment in the evolving landscape of transcatheter aortic valve interventions.

## Early Intervention in Asymptomatic Severe Aortic Stenosis: Rationale and Current Evidence

Current professional societal guidelines recommend a watchful waiting strategy in patients with significant AS and normal left ventricular (LV) function.^1^^,^^2^ However, this strategy appears to be challenged by several observations (Table 1). Despite the historically carried assumption of an intrinsically low risk of asymptomatic significant AS, the risk of sudden cardiac death is higher in such patients than in the general population.^3^ In AS, sustained pressure overload may lead to structural myocardial changes with myocyte apoptosis followed by progressive intramyocardial fibrosis,^4^ even in the absence of symptoms, and it is therefore associated with a worse outcome that may not be reversible despite a successfully performed aortic valve replacement (AVR). Furthermore, delaying intervention may expose the patient with such structural changes to increased perioperative risk if AVR is ultimately required, as well as greater long-term risks of arrhythmia and heart failure. These observations incited clinical investigations of whether early AVR might be beneficial as compared with a “watchful waiting” strategy in these patients. Several observational studies^5^, ^6^, ^7^, ^8^, ^9^ have consistently demonstrated the benefit of the early SAVR approach, mainly driven by improved survival and a lower risk of heart failure hospitalizations. Yet observational studies and subsequent meta-analyses^10^^,^^11^ are exposed to case-selection bias, reliance on purely anamnestic symptom evaluation, and incomplete patient data. Several studies have also included the AVR procedure as a component of the composite outcome but did not detail the number of unoperated patients who developed symptoms prior to SAVR, the number of patients who declined surgery, or the number of patients who were denied SAVR owing to unacceptable risk.

**Table 1:** Pro and contras for AVR in asymptomatic severe aortic stenosis

Pro	Contra
Prevention of sudden cardiac death	Operative mortality
Decrease in morbidity/mortality due to the delayed AVR	Prosthetic valve-related morbidity/mortality
Prevention of structural and functional left ventricular impairment	The earlier the intervention, the higher the probability of requiring future reintervention for bioprosthetic valve

AVR, aortic valve replacement.

Lately, 2 randomized trials have evaluated the role of early surgery for asymptomatic very severe/severe AS and normal LV systolic function—the RECOVERY and AVATAR Trials.^12^^,^^13^ Although appearing similar, they differ in methodological characteristics and should be considered complementary (Table 2). The RECOVERY trial included prevalent population of patients with bicuspid etiology of AS. The conservative arm was managed per current guidelines; however, the study did not utilize systematic exercise testing or biomarker assessment to confirm asymptomatic status at inclusion, with uncertainties about truly asymptomatic status in patients presenting with severe AS with transvalvular velocity exceeding 5 m/s.^12^ The primary endpoint included a composite of operative mortality and death from cardiovascular causes during the entire follow-up period. At follow-up, only 1 patient had died (1%) in the treatment arm vs. 11 (15%) in the conservative management arm [hazard ratio 0.09; 95% CI 0.01-0.67; *p* = 0.003].

**Table 2:** Principal comparative characteristics of Avatar and Recovery trials

	Avatar trial	Recovery trial
Features	Aortic valve area ≤1 cm^2^ with peak aortic jet velocity >4 m/s or a mean transaortic gradient ≥40 mm Hg Asymptomatic status confirmed by negative ET in each patient Mainly degenerative etiology (84%) LVEF >50% Mean follow-up 32 mo	Aortic-valve area ≤0.75 cm^2^ with a peak aortic jet velocity ≥4.5 m/s or a mean transaortic gradient ≥50 mm Hg ET not required Mainly bicuspid etiology (61%) LVEF >50% Mean follow-up 6.1 y
Main findings	Early surgery reduced a composite of all-cause death, acute myocardial infarction, stroke, or unplanned hospitalization for heart failure compared with conservative treatment Hazard ratio, 0.46 [95% CI, 0.23-0.90]; *p* = 0.02	Primary composite end-point of cardiovascular death and operative mortality occurred in 1 patient in the early-surgery group (1%) and in 11 of 72 patients in the conservative-care group (15%) Hazard ratio, 0.09 [95%,CI 0.01-0.67]; *p* = 0.003)
Secondary findings	No difference in major bleeding, thromboembolic complications, repeated MACE No difference in cardiovascular mortality	Death from any cause occurred in 5 patients in the early-surgery group (7%) and in 15 patients in the conservative-care group (21%) (hazard ratio, 0.33; 95% CI, 0.12 to 0.90). No perioperative death Incidence of hospitalization for heart failure lower in the early-surgery group vs. the conservative-care group (0% vs. 11%)
Take homes	Support for early SAVR in severe, truly asymptomatic aortic stenosis, in low-risk patients with normal LVEF	Early SAVR resulted in a significantly lower risk of operative mortality or cardiovascular death

ET, exercise testing; LVEF, left ventricular ejection fraction; MACE, major adverse cardiovascular events; SAVR, surgical aortic valve replacement.

The primary findings of the RECOVERY trial were corroborated by outcomes from the multinational AVATAR trial (aortic valve replacement vs. conservative treatment in asymptomatic severe aortic stenosis).^13^ Over a median follow-up of 3 years, patients randomized to early surgery experienced a lower composite end-point of all-cause death, acute myocardial infarction, stroke, or unplanned HF hospitalization (16.6%) compared to the conservative arm (34.7%) (hazard ratio 0.46; 95% CI 0.23-0.9; *p* = 0.02). However, these outcomes were gathered in a different patient population^14^ than the RECOVERY trial cohort. The Avatar trial included primarily patients with degenerative severe AS (83%), whose asymptomatic status was verified by thorough exercise testing.

Despite synergistically positive signals favoring a shift in the clinical approach to asymptomatic severe AS with different etiology, several limitations for both trials are to be considered. One is the relatively low number of included subjects. This was mainly the case for the AVATAR trial; the trial was event-driven and included a lower number of patients than projected due to a higher number of events and a longer follow-up. Second, the RECOVERY trial reported zero perioperative mortality. This is an exceptional surgical outcome, most likely driven by the trial’s rigorous methodology and possibly by prevalence of bicuspid AS, where patients tend to be younger and have fewer comorbidities. On the other hand, the AVATAR trial reported 1.4% perioperative mortality. This appears more realistic given the patients’ profile with degenerative AS. In the AVATAR trial, the difference in primary outcome was mainly driven by all-cause death and heart failure hospitalizations without a difference in cardiovascular mortality as compared to control group. There were also 2 sudden cardiac deaths during the later follow-up in the early surgery group. Though this requires further analyses, as alluded to above, it should be noted that valve replacement does not necessarily offset the risk of sudden death, as sudden cardiac death remains the second most common cause of cardiovascular death in operated patients.^15^^,^^16^

Taken together, while promising and favoring early intervention in asymptomatic significant AS across randomized trials and observational studies, these findings require further validation in larger prospective randomized controlled trials in patients with severe AS and normal LVEF. One expected study to provide their validation is the Easy AS (NCT04204915) trial, whose sample size is set to include more than 2800 patients. In addition to conventional clinical outcome readouts, these trials should also address the impact of early intervention on patient-reported outcomes. Incorporation of such end-points in the trial readouts is supported by data extracted from Partner 2 and 3 studies, which demonstrated an association between the degree of cardiac damage and quality of life after 1 year, irrespective of symptomatic status, such that patients in stages 3 and 4 had the least improvement from baseline to 1 year and those in stage 0 or 1 achieved the greatest improvement.^17^ In this regard, it is of note that in a subset of patients in the AVATAR trial with available cardiopulmonary exercise testing, patients randomized to early SAVR significantly improved their functional capacity after 12 months in comparison to patients who were conservatively followed and operated only after symptom onset.^18^

## What About Low-Flow Severe Aortic Stenosis?

In patients with AS, a low-flow state, defined as a cardiac index <3.0 l/min/m^2^ or a stroke volume of <35ml/m^2^ ^19^ may be present, and it can be associated with either reduced LVEF (i.e., classical low-flow) or preserved LVEF (i.e., paradoxical low-flow). It is also often associated with a low transvalvular gradient, given that the gradient is highly flow-dependent.^20^ Whether the benefits of early surgery shown in the above-mentioned randomized trials can be extrapolated to subgroups of asymptomatic patients with low-flow, low-gradient AS is unknown. These patients are often considered to be at latter stages of the AS disease spectrum and are anticipated to be more often symptomatic. It is uncertain whether patients with “paradoxical” low-flow, low-gradient AS have worse prognosis in comparison to high-gradient AS.^21^^,^^22^ A patient with low-flow, high-gradient AS is a different entity and might fall into either of 2 groups: symptomatic or asymptomatic. Nevertheless, asymptomatic or minimally symptomatic patients with low-flow, high-gradient AS and preserved LVEF have increased mortality during 5-year follow-up.^23^

Until the indication for AVR in truly asymptomatic patients with severe AS and normal LVEF is established, additional parameters could facilitate patient-tailored decisions irrespective of existing flow pattern. They include extracardiac damage assessment, detection of myocardial fibrosis by cardiac magnetic resonance imaging, valvulo-arterial impedance, global longitudinal strain, and most recently, global myocardial work.^24^, ^25^, ^26^, ^27^ Although these parameters are not yet widely adopted in the practice or considered by either European or US Guidelines, their pathophysiological relevance may support their further adoption in clinical decision-making regarding optimal interventional timing in asymptomatic severe AS patients.

## The Clinical Significance of Moderate Aortic Stenosis

Moderate AS is defined as a maximal aortic jet velocity (V_max_) of 3.0-3.9 m/s and/or a mean pressure gradient of 20-39 mm Hg and/or an aortic valve area (AVA) of >1 ≤ 1.5 cm^2^.^3^ Moderate AS is a progressive disease; 1/3 of patients with moderate AS progress to severe AS in a time frame of approximately 2 ​years, and this progression is associated with a higher risk of cardiac events.^28^^,^^29^ As acknowledged by current European Society of Cardiology and American Heart Association/American College of Cardiology guidelines, patients with rapidly progressive AS (>0.3 m/s annually) also have a higher risk for adverse events, and therefore intervention should be considered in such patients when AS becomes severe.^1^^,^^2^ The recognized predictors of rapid AS progression include initial > mild degree of AS, associated LV hypertrophy, New York Heart Association classification of heart failure class III or IV, aortic valve calcification, and certain demographic features such as male gender or Caucasian race.^30^, ^31^, ^32^ With these data being unveiled, the question arises whether this increased risk of an adverse outcome is a sufficient reason to refer patients with moderate AS to AVR.

It is recognized that patients with moderate AS might also have low-flow state, leading to a discrepancy between measured AVA and mean gradient. Patients with low LV stroke volume (stroke volume index ≤35 mL/m^2^) may have lower mean gradient for a given AVA (AVA <1.0 or 1.5 cm^2^). Likewise in case of patients with severe AS, the combination may exist in the setting of both preserved and reduced systolic function,^28^^,^^29^ termed either concordant or discordant moderate AS. Discordant grading is relatively frequent in patients with moderate AS, encompassing up to 1/3 of patients.^33^ The classified LV flow patterns have been recently shown to have prognostic significance in patients with moderate AS.^34^ Patients with normal flow, low-gradient moderate AS have better survival compared with those with “paradoxical low-flow low-gradient AS” and patients with “classical low-flow low-gradient” moderate AS. Yet, their survival is worse compared to patients with concordant moderate AS (mean gradient 20 to 40 mmHg, and AVA >1 ≤ 1.5 cm^2^). Nevertheless, patients with moderate AS have reduced survival irrespective of whether they present with a concordant or discordant moderate AS, and this has been shown consistently across studies and meta-analyses (Table 3). A large observational study including US and Australian patient population demonstrated the hazard ratio for all-cause mortality was similar for moderate as compared to severe AS (1.38 [95% CI, 1.24-1.53] vs. 1.36 [95% CI, 1.17-1.59], respectively).^39^ The relationship was sustained after adjusting for cofounders that may influence the patient’s individual clinical trajectory and prognosis, such as hypertension, diabetes, coronary artery disease and/or subsequent coronary revascularization, chronic kidney disease, and medications.^37^ Factors that are particularly associated with worse survival in moderate AS are alike to those in patients with severe AS and include reduced LVEF, LV hypertrophy, smaller AVA, and impaired right ventricular function.^43^ In fact, 5-year all-cause mortality exceeds 40% in patients with mean gradient between 20 mmHg and 40 mmHg after adjusting for age, gender, left ventricular systolic and diastolic function, and aortic regurgitation.^43^ In agreement, data from the Heart Valve Clinic International Database revealed 78% mortality in moderate AS patients during an 8-year follow-up.^6^ Particularly poor outcome has been observed in patients with moderate AS and impaired LVEF, with more than 60% reaching the combined end-point of heart failure hospitalization or death by 4-year follow-up.^44^ A meta-analysis of 25 observational studies reported an all-cause mortality rate of 9/100 patients/year, with the majority being cardiac deaths.^28^ In the same meta-analysis, moderate AS patients presenting with diabetes, coronary artery disease, LV dysfunction, and AS-related symptoms had a greater risk for all-cause mortality. LV systolic dysfunction was particularly ominous with 4-fold higher all-cause mortality.

**Table 3:** Studies with >100 patients and meta-analysis investigating the impact of valve replacement in symptomatic patients with moderate AS

Study	Design	Inclusion criteria	Number of patients	Mean age	Mean/median follow-up duration	Main result
Rosenheck et al.^35^	Retrospective	Vmax 2.5 - 3.9 m/s	176	58	55 mo	Estimated event-free survival was 60 ± 4% at 5 y
Minners et al.^36^	Prospective	Vmax 3-4 m/s	948	68	3.8 y	Combined aortic valve related events (AVE), defined as aortic valve replacement (AVR) and all-cause death, 50.9% at 5 y
Strom et al.^37^	Retrospective	Pmax 20-39.9 mmHg/Vmean 3.0-3.9 m/s and AVA ≥1.0 cm^2^	11,987	81.8 (US cohort)	5.2 (US cohort) and 4.4 y (Australian cohort)	Risk of death (hazard ratio) of patients with moderate AS vs. those without AS 1.66 (95% CI 1.52-1.80) and 1.37 (95% CI 1.34-1.41), after adjusting for age and sex The increased risk for death and cardiovascular mortality in patients with moderate AS was consistent across LVEF subgroups
Yechooer, et al.^38^	Retrospective	Vmax 3-4 m/s	104	74	22 mo	Event-free survival 15% at 5 y
Delsalle et al.^39^	Prospective	AVA 1-1.5 cm^2^	508	75	47 mo	6 y survival 53%, prior atrial fibrillation and Charlson comorbidity index associated with increased mortality
Lancellotti et al.^6^	Retrospective	AVA 1-1.5 cm^2^	514	68	2.3 y	94% survival at 2 y 89% at 4 y
Samad Z, et al.^40^	Retrospective	Vmax 3-4 m/s MG 25-39 mmHg AVA >1 cm^2^	1090	75	5 y	26% of patients with moderate AS underwent AVR After multivariable adjustment, AVR associated with higher 5-y survival amongst patients with moderate AS
Strange et al.^41^	Retrospective	Vmax 3.0-3.9 m/s MG 20.0-39.9 mmHg AVA >1 cm^2^	3315	74	1208 d	56% mortality at 5 y
Coisne et al.^28^	Meta-analysis		12,143	/	3.7 y	Pooled rates per 100 person-years 9.0 for all-cause death, 4.9 for cardiac death All-cause mortality higher in patients with LVEF <50% normal LVEF: 16.5 and 4.2 per 100 person-years
Stassen et al.^42^	Retrospective	AVA >1.0 and ≤ 1.5 cm^2^	1961	73	50 mo	868 (44%) patients died during median follow-up of 50 mo At multivariable analysis, NYHA class >1 and LVEF <60% associated with increased mortality
Stassen et al.^34^	Retrospective	AVA >1.0 and ≤1.5 cm^2^	1974	73	60 mo	All-cause mortality 64 and 51% in patients with classical low-flow and paradoxical low-flow discordant moderate aortic stenosis

AS, aortic stenosis; AVA, aortic valve area; AVR, aortic valve replacement; LVEF, left ventricular ejection fraction; NYHA, New York Heart Association; MG, mean gradient.

As decision-making is becoming more complex, clinical practice is embracing the growing evidence that dedicated heart valve clinics bring additional value to diagnostics, treatment and risk stratification of both asymptomatic patients with severe AS and patients with moderate AS.^45^^,^^46^ Given the growing notion of poor outcomes in moderate AS and impaired myocardial function, the decision to intervene in moderate AS is increasingly debated. Initial retrospective studies have demonstrated that patients with moderate AS, especially those who were symptomatic and/or with impaired LVEF, have benefited from AVR.^40^^,^^47^ Table 4 summarizes ongoing randomized trials investigating whether early AVR yields benefit on top of optimal medical management in patients with moderate AS (Table 4). All ongoing randomized trials use an exclusively transcatheter approach.

**Table 4:** Ongoing trials in moderate AS

Trial	Number of patients	Design	Type of intervention	Primary outcome	Follow-up duration	Estimated primary completion
Expand TAVR	650	Randomized trial	TAVR	Composite rate of all-cause mortality or unplanned procedure-related or aortic valve related hospitalization	2 y	2026
Progress	750	Randomized trial	TAVR	A composite of death, stroke, and unplanned cardiovascular hospitalization	2 y	2029
TAVR Unload	300	Randomized trial	TAVR	Hierarchical occurrence within efficacy assessment time interval of All-cause death, stroke, heart failure hospitalization	1 y	2023

TAVR, transcatheter aortic valve replacement.

The rationale for intervention in patients with moderate AS and with heart failure symptoms seems clinically reasonable, as heart failure is the essential driver of symptoms and increases susceptibility to poor outcomes.^40^ Patients with reduced systolic LV function are vulnerable to the increased LV afterload due to even moderate AS^48^ and experience a 3-fold increase in mortality as compared to those without AS.^44^ Moderate AS, even if asymptomatic, might also be accompanied by changes in the structure of the myocardium, as was documented with the presence of focal irreversible fibrosis and scar formation by cardiac magnetic resonance.^49^ These structural myocardial changes in patients with moderate AS appear to be predictive of all-cause mortality to the same extent as in patients with severe AS.^49^ The potential to halt or even reverse some of the maladaptive structural and functional cardiac changes that can accompany moderate AS through earlier valve replacement and load reduction may deserve the consideration to test clinically such hypothesis.

## Growing TAVR Adoption: Open Questions and Life-Long Treatment Strategy in AS

TAVR has proven efficient and competitive as an alternative to SAVR across groups of patients with different surgical risk, including low-risk symptomatic AS patients.^50^^,^^51^ Yet, until now, the benefit of early AVR in asymptomatic patients with similarly severe AS and normal LVEF has been demonstrated only for a surgical approach. Among currently ongoing randomized trials, only the Early TAVR trial is testing an exclusively transcatheter femoral approach. However, before TAVR could be implemented as an intervention of choice for all patients with AS, irrespective of age, symptoms, or risk, it will need to demonstrate long-term durability comparable to surgically implanted prosthetic valves. Until then, the whole range of SAVR approaches should be considered, particularly in younger patients, including bioprosthetic/mechanical valves, SAVR with aortic root enlargement, and in prespecified younger patients, the Ross procedure. In the hands of an experienced surgeon, the Ross procedure may be a viable option for carefully selected younger patients who are anatomically suitable, owing to its better survival rates compared to conventional SAVR with both mechanical and bioprosthetic valves.^52^ In younger asymptomatic patients with an anticipated longer life-span and active lifestyle as compared to elderly patients, several TAVR complications, such as the need for reintervention, permanent pacemaker implantation, mild paravalvular regurgitation, or the TAVR stent frame height leading to a possible barrier to future coronary access, are not acceptable (Table 5).

**Table 5:** Open questions in asymptomatic severe aortic stenosis and symptomatic moderate aortic stenosis

Open questions		
Asymptomatic severe AS		Symptomatic moderate AS
*Global questions*	*Procedure and valve related*	1. Whether TAVR in patients with isolated moderate AS offers survival benefit on top of standard-of-care treatment of HF with reduced LVEF? 2. Usefulness of TAVR in patients with moderate AS and HFpEF 3. The role of novel imaging modalities in treatment of patients with moderate AS
1. Establishing further benefit of early AVR in asAS 2. The proposed strategy for long-term treatment in younger (<65), asymptomatic low-risk patients with severe AS Proposed strategy: - SAVR - TAVR (if needed) - TAVR (if needed)	1. The challenge of future coronary access	
	2. Durability of TAVR valve	
	3. The prognostic importance of: patient-prosthesis mismatch, need for permanent pacemaker and mild PVL	
*Next steps*		*Next steps*
1. Ongoing trials evaluating early AVR vs. watchful waiting strategy (NCT04204915; NCT03042104; NCT03972644; NCT03094131) 2. Ongoing trials evaluating TAVR valve durability throughout the long-term follow-up (up to 10 y follow-up) in low-risk patients (Partner 3,Evolut-low risk trial extended follow-up) 3. The ongoing Alliance Trial (NCT05172960) 4. Warranted randomized trial of SAVR vs. TAVR in asymptomatic middle-aged severe AS patients with normal LVEF		1. Several ongoing trial evaluating TAVR in symptomatic patients with moderate AS (see Table 4)

AS, aortic stenosis; asAS, asymptomatic aortic stenosis; AVR, aortic valve replacement; HF, heart failure; HFpEF, heart failure with preserved ejection fraction; LVEF, left ventricular ejection fraction; PVL, paravalvular leak; SAVR, surgical aortic valve replacement; TAVR, transcatheter aortic valve replacement.

Hence, we face the question of how to manage younger AS patients over a long-term period with life-span of >15 years and provide them with the highest possible assurance of durable treatment outcome. Surgery is still considered the standard of care for patients <75 years of age. According to current guidelines,^1^^,^^2^ in patients younger than 50 years, a mechanical valve is indicated, while in patients between 50-65 years, an individualized approach is recommended, with consideration of individual patient factors and informed shared decision-making. Thus, patients <50 years should preferably receive a mechanical valve, and consequently, there should be no room for debate about a long-term treatment strategy. Nevertheless, these patients should be served with rigorous follow-up and control of anticoagulation.

In patients above 50 years and less than 80, several strategies are possible to consider. In the context of encouraging TAVR outcomes, the majority of patients and some physicians may be inclined to opt initially for less invasive TAVR as a treatment of choice, including low-risk patients. Regardless of whether a biological surgical or TAVR valve is implanted, ultimately these valves will start to degenerate and will need replacement. In the case of surgical valves, the durability will typically reach 10-15 years, while TAVR valve durability has been demonstrated up to 8 years so far in a limited number of patients.^53^ Of note, the risk of structural valve deterioration was shown to be lower after TAVR in comparison to surgical valves in patients with low-to-moderate surgical risk for 5 years of follow-up.^53^^,^^54^ However, these data should be interpreted carefully, as structural valve degeneration after SAVR is increased in situations of patient-prosthesis mismatch. The patient-prosthesis mismatch occurs typically in ≈10% of patients.^55^ However, it has been reported that 40% of surgical patients experience early surgical valve degeneration.^53^ In addition, the clinical impact of patient-prosthesis mismatch in patients undergoing TAVR is not entirely clear.^56^ Life expectancy is another variable influencing the choice of treatment strategy, as suggested by current European guidelines. It is, however, region- and country-specific and difficult to predict for an individual patient.

Patients undergoing AVR in their early 60s may conceivably require additional valve replacement during their lifetime.^57^ This potentially might lead to the “Matryoshka doll” effect if only TAVR were considered. In such a scenario, several TAVR valve-in-valves implanted into each other would lead to too small an effective orifice area, insufficient for adequate blood pumping in the long-term. This may also lead to increased risk of coronary obstruction and jeopardized coronary access, especially in the setting of acute coronary syndrome when percutaneous coronary intervention (PCI) is time-sensitive. It is then common sense that SAVR should remain a first choice as being safer and likely more efficient at younger age. This is also supported by recent findings reporting excessive surgical mortality in SAVR after a previous TAVR procedure, ranging from 20% to 40%.^58^^,^^59^ Though new TAVR valves undergo further refinements with each new generation, their durability or adverse impact regarding, for instance, future coronary access, is being evaluated in the new Alliance trial (NCT05172960) using the newest, fourth generation of a balloon-expandable valve. With evolving designs of TAVR prosthetic valves, a randomized trial comparing TAVR with SAVR in low-risk, middle-aged, asymptomatic patients with normal LV systolic function might be an adequate approach to address this clinically critical question. From the caregiver perspective, health economic analysis of such an approach addressing the procedure-related cost-effectiveness in the long term is also relevant.

Finally, aortic valve interventions in patients with concomitant coronary artery disease require further attention. One aggravating fact is that coronary artery disease might be masked in patients with hemodynamically significant AS.^60^ The prevalence of coronary artery disease in AS patients is high, ranging between 40 and 80%, depending on age and surgical risk.^50^^,^^51^ In younger, low-risk patients, especially those with more complex coronary artery disease, the benefit of concomitant SAVR and coronary artery bypass grafting is established and recommended by guidelines.^1^^,^^2^ On the other hand, the benefits and optimal timing of PCI in TAVR candidates are still a matter of ongoing debate and research. A recent study found no benefit of a “PCI first” strategy in patients undergoing TAVR. However, it demonstrated higher 5-year all-cause mortality for TAVR patients with coronary artery disease, with increased risk correlated with coronary artery disease complexity.^61^ Nevertheless, in the ACTIVATION trial, mortality and rehospitalization rates at 1 year were similar between PCI and no PCI prior to TAVR, and PCI resulted in higher bleeding complications.^62^ Hence, more robust data are needed regarding the role of PCI in TAVR candidates. Until then, SAVR and coronary artery bypass grafting remain the most optimal strategies for these patients, especially for more complex coronary artery disease.

## Conclusion

Symptomatic AS is an ominous disease requiring timely valve intervention. Growing evidence indicates a detrimental clinical impact of asymptomatic significant AS and even of moderate AS in symptomatic patients. While 2 randomized trials in the setting of asymptomatic AS provide preliminary support for early intervention in such patients, ongoing studies are addressing the impact of such interventions in moderate AS. After the TAVR procedure has emerged as a viable alternative to SAVR across the whole spectrum of symptomatic AS patients with different surgical risks, new directions in clinical research have been set to address how to optimally manage other patient groups. This requires carefully designed trials to rigorously address questions related to the durability of interventional strategy balancing the consequence of the primary choice with the patient's future life-span.

## Funding

The authors have no funding to report.

## Disclosure Statement

Marko Banovic, Bernard Iung, Jozef Bartunek, Wojtek Wojakowski are investigators in the Avatar trial; Jozef Bartunek receives consultancy fees from Occlutech (unrelated to the topic of the manuscript). The other author had no conflicts to declare.
